# Peripartum HIV infection in very low birth weight infants fed ‘raw’ mother’s own milk

**DOI:** 10.4102/sajhivmed.v20i1.912

**Published:** 2019-06-19

**Authors:** Melantha Coetzee, Suzanne D. Delport

**Affiliations:** 1 Department of Paediatrics and Child Health, Steve Biko Academic Hospital, Pretoria, South Africa; 2 Faculty of Health Sciences, University of Pretoria, Pretoria, South Africa; 3 Division of Neonatology, Department of Paediatrics, Kalafong Provincial Tertiary Hospital, Pretoria, South Africa

**Keywords:** HIV, Prevention of mother-to-child transmission, Mother-to-child transmission, Very low birth weight, Peripartum transmission, Mother’s own milk, Raw breast milk, Nevirapine

## Abstract

**Background:**

HIV-exposed very low birth weight (VLBW) infants (≤ 1500 g) are considered at high risk of peripartum mother-to-child HIV transmission (MTCT). In the past, they received formula to prevent breast milk related HIV transmission. This denied them the benefits of breast milk, thus exposing the infant to the risk of necrotising enterocolitis (NEC). From 2010, ‘raw’ mother’s own milk (rMOM) has been recommended for term infants whose mothers’ received antenatal antiretroviral therapy (ART). At the same time, the infant received antiretroviral (ARV) prophylaxis as per the National Prevention of MTCT programme.

**Objectives:**

To determine the cumulative incidence of peripartum HIV infection by 4–6 weeks of age in HIV-exposed VLBW infants, who received rMOM and infant ARV prophylaxis.

**Method:**

A retrospective, observational audit over 3 years at a single institution was undertaken. The study population comprised HIV-exposed VLBW infants who received both nevirapine prophylaxis and rMOM from birth until discharge. A positive HIV-PCR by 4–6 weeks of life was used to confirm maternal to infant HIV transmission.

**Results:**

Of the 80 eligible infants admitted between 2010 and 2013, 63 (79%) were exposed to antenatal ART. Seventy-eight (97.5%) tested HIV-PCR negative at 4–6 weeks. Of the two infants who tested positive, both presented with features of an acute HIV infection. The absence of MTCT in the remaining 78 infants given ARV prophylaxis and rMOM suggests that rMOM is an unlikely source of infection in the two infected infants.

**Conclusion:**

rMOM, in the presence of infant prophylaxis, was a safe feeding option for HIV-exposed VLBW infants. It should be strongly considered for these infants, as rMOM likely provides additional maternal and child benefits.

## Introduction

South Africa is the global epicentre of the human immunodeficiency virus (HIV) pandemic, with an antenatal prevalence of 31%.^1^ Human immunodeficiency virus-infected women are at increased risk of delivering low birth weight and/or preterm infants^2^ and of transmitting infection to their infants. HIV transmission is higher in the absence of maternal antiretroviral therapy (ART);^3,4,5,6^ greater with higher maternal viral load; greater with worsening immunosuppression (low CD4 count);^3,4,6^ and is increased in the presence of maternal infections such as tuberculosis and sexually transmitted disease.^7^ Increased permeability of the intestinal mucosal barrier in preterm infants further increases the risk of mother-to-child transmission (MTCT).^4,6^ These infants are also at risk of necrotising enterocolitis (NEC) if formula feeds are administered in an effort to reduce MTCT.^8^ Mother’s own milk (MOM) is crucial to the survival of preterm infants.^9^ The risk and benefit to VLBW HIV-exposed infants receiving prevention of mother-to-child transmission (PMTCT) interventions and ‘raw’ MOM (rMOM) is unclear. Preterm and VLBW infants receiving rMOM have fewer infections and less NEC.^8,9^ Whether this is true for HIV-exposed preterm and VLBW infants has not been studied.

PMTCT programmes in South Africa before 2002 were limited because of governmental AIDS denialism and concerns related to ART toxicity.^10^ In 2002, a Constitutional Court ruling mandated rolling-out of a PMTCT programme.^11^ Reports of MTCT of HIV in VLBW infants followed,^12,13,14,15,16,17^ but these failed to determine the safest feeding choice in such infants. The feeding regimens in these studies included exclusive formula feeding (EFF) in line with the National PMTCT programme at the time,^18,19^ exclusive donor breast milk (DBM) (holder pasteurised) and heat-treated MOM (Pretoria pasteurised or flash-heated).^20,21^

Annual studies reporting on MTCT in infants ≤ 1500 g from 2005 to 2007 noted HIV transmission rates of 14.9%,^12^ 19.0%^13^ and 10.0%,^14^ respectively. At this time, the implementation of ART for PMTCT in South Africa was inconsistently applied (Table 1). Subsequently (2008–2015), MTCT declined from 7.6% to 0.0%^15,16,17^ when PMTCT became the standard of care (Table 1). This included improved infant regimens at and post-delivery^18,19,22,23,24^ and the provision of maternal combination ART (cART) to pregnant and breastfeeding women.^23^ No infants included in these studies (*n* = 289) received rMOM from birth.^15,16,17^

**TABLE 1:** South African studies reporting on human immunodeficiency virus transmission by postnatal age in infants ≤ 1500 g birth weight.

Year Hospital	Birth weight (*n*)	Maternal ART	Infant regimen	Infant feeding	MTCT of HIV
**2003–2005** Tygerberg^12^	< 1500 g (141)	sdNVP during labour or AZT from 34 weeks gestation with sdNVP during labour: 72%	sdNVP or sdNVP+AZT: 99%	Heat-treated EBM/DBM/EFF	14.9% by 14 weeks
**2006–2007** Chris Hani Baragwanath^13^	< 1500 g (26)	cART: 1% NVP before delivery: 36%	sdNVP: 73%	Not documented	19% by 6 weeks (95% CI: 7% – 40%)
**2006–2007** Kalafong^14^	≤ 1500 g (83)	sdNVP: 37% cART: 13% No ART: 50%	sdNVP: 100%	Heat-treated EBM/EFF	10% by 6 weeks
**2007–2008** Tygerberg^15^	≤ 1500 g (185)	cART: 17% AZT/NVP before delivery: 28% AZT/NVP during labour: 43% No ART: 15%	AZT: 100%	Heat-treated EBM/DBM/EFF	7.6% by ≥ 2 weeks
**2010–2011** Groote Schuur^16^	≤ 1000 g (37)	Some ART: 72% (ART >1 month before delivery: 44%)	NVP or sdNVP+AZT: 100%	Heat-treated EBM/DBM/EFF	2.7% by 6 weeks (95% CI: 0.7% –14.1%)
**2014–2015** Groote Schuur and New Somerset^17^	< 1500 g (67)†	ART >1 month before delivery: 72% ART <1 month before delivery: 9% No ART: 13%	NVP+AZT: 100%	Heat-treated EBM until infant can breastfeed/DBM/ EFF	0% by 6 weeks

ART, antiretroviral therapy; AZT, azidothymidine; DBM, donor breast milk; EFF, exclusive formula feeding; cART, combination antiretroviral therapy; NVP, nevirapine; sdNVP, single dose nevirapine; PCR, polymerase chain reaction; EBM, expressed breast milk.

† Number of infants calculated from the statement by the authors that 87% of the cohort that was negative at birth (*n* = 77) was tested at 6 weeks of age.

Most HIV-exposed preterm infants prior to initiation of modern PMTCT programmes received EFF, as did their term counterparts.^18,19^ Subsequently, the safety of heat-treated expressed MOM was confirmed,^20,21^ and this became the feeding choice for hospitalised preterm infants in many facilities. Although affordable,^20,21^ heat-treated MOM is labour-intensive in a hospital setting and for mothers after discharge. Also, it remained unclear whether non-HIV-related benefits of MOM were lost through the heat treatment.

## Study objective

A retrospective, observational audit was undertaken to determine the cumulative incidence of peripartum HIV transmission at 4–6 weeks of age in HIV-exposed VLBW infants, who received infant prophylaxis according to the National PMTCT programme of 2010^19,22^ and ‘predominantly’ raw MOM (prMOM) (see Study Population section for the definition of prMOM).

## Material and methods

### Setting

This study was performed in the Neonatal Unit of Kalafong Provincial Tertiary Hospital, South Africa. The neonatal unit comprises 30 beds, which include 6 neonatal intensive care beds. There are approximately 5900 deliveries per year at Kalafong Provincial Tertiary Hospital, with a maternal HIV prevalence of approximately 18%.

### Study population

The study population was identified from ward registers. The start of this study, 01 March 2010, coincided with the implementation of the National 2010 PMTCT guideline. Records of patients admitted to the unit in the subsequent 36 consecutive months were audited. Breast milk was prescribed to all infants admitted to the neonatal unit and supplemented by DBM if MOM was insufficient.

Infants were included if they were HIV-exposed, that is, born to an HIV-positive mother and received prMOM, that is, at least two-thirds of the total enteral volume, over the study period. The remaining one-third enteral fluid volume was DBM and was given with infant antiretroviral prophylaxis. The latter was daily nevirapine from birth until at least 6 weeks of age. A surveillance HIV-PCR was checked at 6 weeks as per the 2010 National PMTCT programme.^19,22^ (Table 2).

**TABLE 2:** Maternal antiretroviral therapy, infant prophylaxis and feeding regimens recommended by the South African national prevention of mother-to-child transmission programme of 2010 for human immunodeficiency virus-positive women during pregnancy and after delivery and their human immunodeficiency virus-exposed infants.

Variable	PMTCT programme	
	2010 (1st edition)^19^	2010 (2nd edition)^22^
**Maternal ART regimen**		
CD4 > 350 cells/mm^3^	Dual therapyn†	Dual therapy†
CD4 ≤ 350 cells/mm^3^ or stage 3 or 4 HIV	Lifelong cART‡	Lifelong cART‡
**Infant prophylaxis**		
EFF	NVP for 6 weeks	NVP for 6 weeks
Breastfeeding without maternal lifelong cART	NVP until 1 week post-cessation of breastfeeding	NVP until 1 week post-cessation of breastfeeding
Breastfeeding with maternal lifelong cART	NVP for 6 weeks	NVP for 6 weeks
**Infant feeding regimens**	EFF if the AFASS criteria are met Exclusive breastfeeding if AFASS criteria are not met	Exclusive breastfeeding for all infants

AFASS, acceptable, feasible, affordable, safe, sustainable; AZT, azidothymidine; cART, combination antiretroviral therapy; EFF, exclusive formula feeding; FTC, emtricitabine; NVP, nevirapine; TDF, tenofovir.

† Dual therapy: AZT from 14 weeks gestation, followed by three-hourly AZT and single dose NVP during labour, with a single dose of TDF and FTC after delivery (ART discontinued after delivery).

‡ Lifelong cART: TDF + lamivudine/FTC + NVP/efavirenz (continued after delivery).

Deviations from the surveillance regimen^19,22^ were indicated in some infants. These included HIV-PCR testing at ‘non-routine’ times, for example, within 72 h after birth to diagnose congenital infection; before 6 weeks of age in the event of clinical signs suggestive of HIV infection; and should discharge occur before 4 weeks of age to minimise the number who might not return and so be lost to follow-up. All HIV-PCR test results were accessed from patient files and the National Health Laboratory Service (NHLS) database.

### Definitions

#### Congenital infection

*In utero* acquisition of HIV (congenital infection) was diagnosed when an HIV-PCR was positive within 72 h of birth.

#### Peripartum infection

The MTCT of HIV during the peripartum period was defined as HIV acquisition during labour, delivery or while receiving prMOM and nevirapine (NVP) prophylaxis. It was diagnosed when an HIV-PCR test was negative within 72 h after birth yet positive at 4–6 weeks. In the absence of an early HIV-PCR, peripartum infection was excluded if the HIV-PCR was negative at 4–6 weeks.

### Exclusion criteria

**FIGURE 1:**
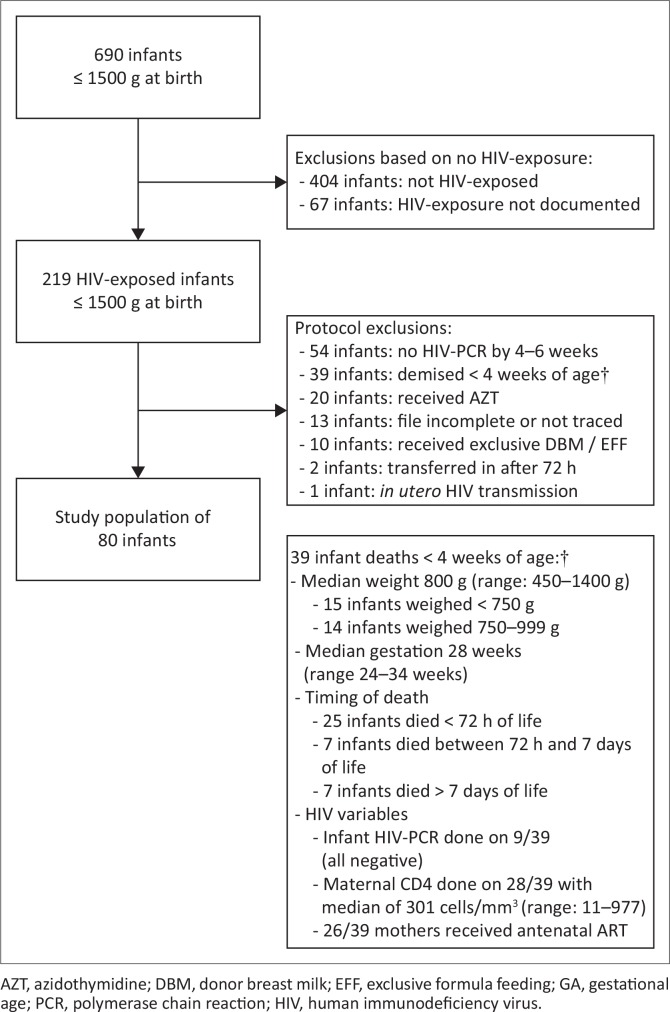
Flow chart detailing exclusions.

Infants were excluded if death occurred before 4 weeks of age, azidothymidine (AZT) was used as PMTCT, feeds were exclusively DBM or EFF, no HIV-PCR result was available at 4–6 weeks of age, admission to the neonatal unit occurred after 72 h of life, HIV was deemed to have been acquired *in utero* and clinical records were missing or incomplete (Figure 1).

### Background

#### Infant feeding regimens

Although free tins of formula were provided for HIV-exposed infants who complied with the acceptable, feasible, affordable, safe and sustainable (AFASS) feeding criteria,^21^ breastfeeding was officially adopted in August 2011 (Tshwane Declaration) as the feeding regimen of choice for all infants, including those who were HIV-exposed^25^ as breast milk was shown to be safe in these infants provided they received PMTCT^26^ (Table 2).

#### Maternal antiretroviral therapy regimens

The National PMTCT programme of 2010 used the maternal CD4 count and HIV staging to determine maternal antenatal and postnatal ART regimens^19,22,27^ (Table 2).

#### Exposure of infants to maternal antenatal antiretroviral therapy

Duration of exposure to maternal antenatal ART (lifelong cART or dual therapy) was defined, for the purpose of this study, as optimal (≥ 4 weeks), suboptimal (< 4 weeks) or no ART.

#### Infant prophylaxis

All infants were initiated on NVP after delivery, which was continued for at least 6 weeks. A weight-based dosing regimen, as recommended by the World Health Organization,^28^ was adopted for infants weighing < 1800 g: 2 mg/kg daily for the first 2 weeks of life and 4 mg/kg thereafter.^29^

### Data analysis

Statistical analysis was performed using Stata statistical software 2017 (Release 15.1, StataCorp LLC, College Station, US). Continuous data were expressed as medians and ranges and categorical data as frequencies and percentages. Cumulative incidence and 95% confidence intervals were determined using Poisson regression.

## Ethical consideration

The study protocol was approved by the Ethics Committee of the Faculty of Health Sciences of the University of Pretoria, South Africa (Ethics approval number 351/2013).

## Results

Over the 3-year period from 01 March 2010 to 28 February 2013, 3790 newborn infants were admitted to the Neonatal Unit, of whom 3774/3790 (99.58%) had a documented birth weight. Of these infants, 690/3774 (18.28%) had a birth weight ≤ 1500 g; 404/690 (58.55%) of these were not HIV-exposed; 219/690 (31.74%) were HIV-exposed; and a further 67/690 (9.71%) had no documented maternal HIV test result.

### Study population and exclusions

The HIV-exposed infants (219/690) were the preliminary study population. As per protocol, 139/219 infants were excluded, as shown in Figure 1. A sample of 80/219 HIV-exposed infants remained and formed the final study population. The details of the 39 infants who died and were consequently excluded from the study are also shown in Figure 1.

### Maternal data of the study population

The maternal population totalled 72 mothers: 8/80 infants were four twin pairs. Of these mothers, 61/72 (84.72%) received antenatal care during pregnancy. All had non-reactive rapid plasma reagin (RPR) tests for syphilis, while 7/72 (9.72%) received treatment for tuberculosis (TB). The demographics of the HIV-infected mothers is shown in Table 3. The median CD4 count was 272 cells/mm^3^ (range 8–1097 cells/mm^3^), and the median HIV viral load was 7191 copies/mL (range 0–68 952 copies/mL). It should be noted that only 77.78% (56/72) of HIV-infected women were receiving ART during their pregnancy.

**TABLE 3:** Maternal human immunodeficiency virus characteristics

Variable	All mothers *N* = 72 (%)		MTCT absent *N* = 70 (%)		MTCT present *N* = 2 (%)	
	*n*	%	*n*	%	*n*	%
**Timing of HIV diagnosis**	**72**	**-**	**70**	**-**	**2**	**-**
Before pregnancy	7	9.72	7	10.0	-	-
During pregnancy	15	20.83	15	21.42	-	-
At delivery	5	6.94	4	5.71	1	50.0
Post-delivery	3	4.17	2	2.86	**-**	**-**
Not documented	42	58.33	42	60.0	1	50.0
**CD4 count (cells/mm^3^)**	**71**	**-**	**69**	**-**	**2**	**-**
≤ 350	42	59.15	41	59.42	1	50.0
> 350	29	40.85	28	40.58	1	50.0
**HIV VL (copies/mL)**	**72**	**-**	**70**	**-**	**2**	**-**
< 1000	3	4.17	3	4.29	**-**	**-**
≥ 1000	9	12.50	8	11.42	1	50.0
Not determined	60	83.33	59	84.29	1	50.0
**Antenatal ART**	**72**	**-**	**70**	**-**	**2**	**-**
Any	56	77.78	56	80.0	**-**	**-**
Lifelong cART	33	45.83	33	47.14	**-**	**-**
Dual therapy	23	31.94	23	32.86	**-**	**-**
None	15	20.83†	13	18.57	2	100
Not documented	1	1.39	1	1.43	**-**	**-**
**Duration of antenatal ART**	**56**	**-**	**56**	**-**	**0**	**-**
≥ 4 weeks	33	58.92	33	58.92	**-**	**-**
Lifelong cART	25	44.64	25	44.64	**-**	**-**
Dual therapy	8	14.29	8	14.29	**-**	**-**
< 4 weeks	11	19.64	11	19.64	**-**	**-**
Not documented	12	21.43	12	21.43	**-**	**-**
**Postnatal lifelong cART**	**72**	**-**	**70**	**-**	**2**	**-**
Continued after delivery	33	45.83	33	47.14	**-**	**-**
Initiated after delivery	5	6.94	4	5.71	1	50.0
Not indicated	33	45.83	32	45.71	1	50.0
Not documented	1	1.39	1	1.43	**-**	**-**

ART, antiretroviral therapy; HIV, human immunodeficiency virus; VL, viral load; MTCT, mother-to-child HIV-transmission.

† One twin pair.

### Clinical characteristics of the study population (*n* = 80)

**FIGURE 2:**
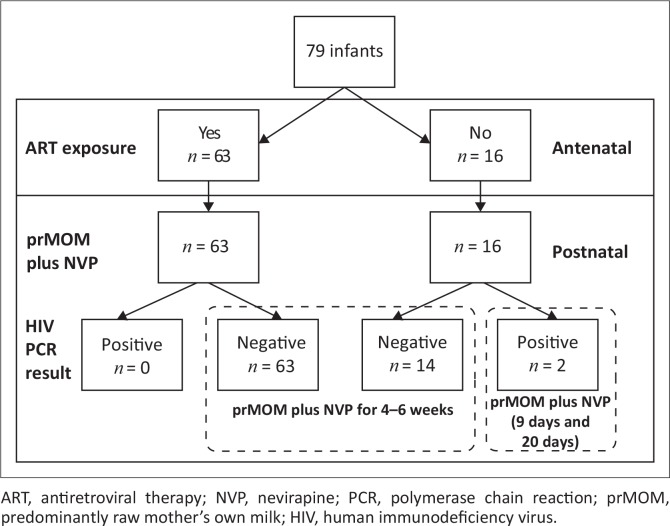
The safety of ‘raw’ mother’s own milk in human immunodeficiency virus-exposed very low birth weight infants.

Infant median weight was 1130 g (range 510–1500 g) and the median gestational age was 30 weeks (range 25–38 weeks). Additional data are shown in Table 4. During pregnancy, only 78.75% (63/80) of infants had any antenatal ART exposure. Twenty per cent had no exposure. The antenatal ART exposure of one infant was undocumented. After delivery, all infants (*n* = 80) received both postnatal NVP and prMOM until discharge (Figure 2).

**TABLE 4:** Infant characteristics.

Clinical features	All infants *N* = 80 (%)		HIV-exposed uninfected infants *N* = 78 (%)		HIV-infected infants *N* = 2 (%)	
	*n*	%	*n*	%	*n*	%
**Pregnancy**	**80**	-	**78**	**-**	**2**	**-**
Singleton	60	75.0	58	74.36	2	100
Multiple (twin)	20	25.0	20	25.64	-	-
**Gender**	**80**	**-**	**78**	**-**	**2**	**-**
Female	46	57.50	45	57.69	1	50.0
Male	34	42.50	33	42.31	1	50.0
**Mode of delivery**	**78**	**-**	**77**	**-**	**1**	**-**
Caesarean section	43	55.13	43	55.84		
Vaginal delivery	30	38.46	29	37.66	1	100
BBA	5	6.41	5	6.49	-	-
**Place of delivery**	**79**	**-**	**78**	**-**	**1**	**-**
Inborn	69	87.34	68	87.18	1	100
Outborn (including BBA)	10	12.66	10	12.82	-	-
**Amniotic membranes**	**49**	**-**	**49**	**-**	**0**	**-**
Intact	29	59.18	29	59.18	-	-
Ruptured < 24 h	9	18.37	9	18.37	-	-
Ruptured ≥ 24 h†	11	22.45	11	22.45	-	-
**Placental histology**	**16**	**-**	**16**	**-**	**0**	**-**
Acute chorioamnionitis	9	56.25	9	56.25	-	-
**Tuberculosis exposed**	**8**	**-**	**8**	**-**	**0**	**-**
Received treatment	3	37.50	3	37.50		
**ART**						
**Exposure to antenatal ART**	**80**	**-**	**78**	**-**	**2**	**-**
Yes	63	78.75	63	80.77	-	-
No	16	20.0	14	17.95	2	100
Not documented	1	1.25	1	1.28	-	-
**Exposure to ≥ 4 weeks antenatal ART**	**49**	**-**	**49**	**-**	**0**	**-**
Yes	37	75.51	37	75.51	-	-
Lifelong cART	27	55.10	27	55.10	-	-
Dual therapy	10	20.41	10	20.41	-	-
**Exposure to postnatal maternal cART**	**80**	**-**	**78**	**-**	**2**	**-**
Yes	40	50.0	40	51.28	-	-
No	39	48.75	37	47.44	2	100
Not documented	1	1.25	1	1.28	-	-
**Infant prophylaxis**	**80**	**-**	**78**	**-**	**2**	**-**
Yes	80	100	78	100	2	100
**Feeding**	**80**	-	**78**	**-**	**2**	**-**
prMOM	80	100	78	100	2	100
Supplemental DBM‡	21	26.25	21	26.92	-	-
**HIV-PCR**	**80**	**-**	**78**	**-**	**2**	**-**
Negative	78	97.50	78	100	-	-
Positive	2	2.50	-	-	2	100

BBA, born before arrival; cART, combination antiretroviral therapy; DBM, donor breast milk; NVP, nevirapine; PCR, polymerase chain reaction; prMOM, predominantly raw mother’s own milk.

† Prolonged ROM is defined as ROM ≥ 24 h.

‡ Less than one-third of the total enteral intake.

#### Postnatal prophylaxis

All 80 infants were hospitalised after birth and received supervised daily NVP until discharge. The majority of infants (67/80) received the first dose of NVP within 24 h of birth. Seven infants received it after 24 h (range 30–84 h). The timing of the first dose was not recorded for six infants. Just more than half of the infants (41/80) received NVP at the recommended daily dose of 2 mg/kg, with doses varying between 2 mg/kg and 10 mg/kg (median 2 mg/kg), but never exceeding a total daily dose of 10 mg.

In addition to NVP, 40/80 (50.0%) infants were also exposed to maternal lifelong cART during breastfeeding.

#### Feeding regimen with mother’s own milk

In keeping with the exclusive breastfeeding policy of the Neonatal Unit, MOM was prescribed for all infants after birth. Three-quarters, viz. 59/80 (73.8%) of infants, received rMOM exclusively until discharge. The remainder, viz. 21/80, required supplementation with DBM. The median proportion of the volume of DBM intake of the 21/80 infants was 8.96% (range 1.67% – 33.33%) of the total enteral intake. No infant received formula milk.

### Mother-to-child transmission of human immunodeficiency virus

A definitive HIV-PCR was performed on 78/80 infants by 4–6 weeks of age to rule out peripartum acquisition of HIV; *n* = 45/78 infants tested negative before discharge; 33/78 infants tested negative at follow-up, viz. 67/78 at 6 weeks, 7/78 at 5 weeks and 4/78 at 4 weeks of age. Two infants tested positive: one on day 9 and the other on day 20 of life. These HIV-PCR tests were performed earlier because clinical signs suggested active HIV infection. Human immunodeficiency virus infection in these two infants was confirmed with a second (follow-up) HIV-PCR, and they were initiated on lifelong cART. Neither had an HIV-PCR within 72 h of birth, so *in utero* HIV infection cannot be excluded. Their birth weights were 1120 g and 1400 g, respectively. Both were on NVP prophylaxis and prMOM from birth. Neither had been exposed to maternal ART before birth. Fourteen (14/78) other HIV-exposed but uninfected (HEU) infants remained uninfected despite the absence of exposure to maternal ART prior to birth (Table 4).

**FIGURE 3:**
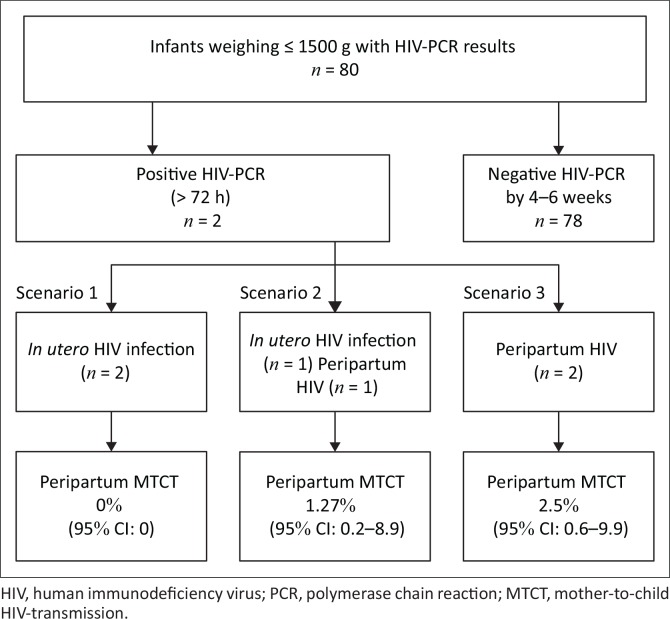
Three scenarios related to cumulative incidence of peripartum human immunodeficiency virus transmission by 4–6 weeks.

The time of HIV acquisition in the two infected infants (namely *in utero* as opposed to peripartum) could not be determined with certainty. Therefore, the cumulative incidence of peripartum HIV transmission by 4–6 weeks of age in the study population is expressed as ranging between 0% and 2.5%. It would be 0% had both these infants acquired HIV *in utero*, 1.27% (95% CI: 0.2–8.9) had one infant acquired HIV during the peripartum period, and 2.5% (95% CI: 0.6–9.9) had both acquired HIV during the peripartum period (Figure 3).

## Discussion

The cumulative incidence of peripartum HIV infection in VLBW infants by 4–6 weeks receiving the National PMTCT programme of 2010 ^19,22^ in addition to prMOM is 2.5%. This is comparable to the MTCT of 2.7% in a more vulnerable cohort of extremely low birth weight South African infants at 6 weeks.^16^ This cohort, studied when the National PMTCT programme of 2010^19,22^ was operational, did not receive rMOM.^16^ The VLBW infants in our study received prMOM and its additional nutritional and immunological advantages with seemingly no increased risk of MTCT. This finding lends support for the contention that exclusive breastfeeding of HIV-exposed infants^3^ and their VLBW counterparts is safe in the context of antiretroviral prophylaxis.

As previously documented, 690 of the 3774 infants (18.28%) admitted to the Neonatal Unit at Kalafong Provincial Tertiary Hospital were VLBW, 219 being HIV-exposed. Their risk of being already HIV-infected at the time of birth (*in utero* HIV acquisition) or acquiring the infection during the peripartum period could be minimised by timeous antenatal as well as postnatal maternal ART. Providing the infant with additional ART prophylaxis after birth should further reduce the risk of peripartum HIV transmission, particularly via breast milk. This directive was mandated by the National PMTCT programme of 2010,^19,22^ at the time of this study, however was not reliably applied especially during the antenatal period. At least 15/72 women received no ART during pregnancy, which increased the risk of *in utero* and peripartum infection in 16 infants in the study population (one mother had twins). Only two of these 16 infants acquired HIV infection, while none of the infants exposed to antenatal ART acquired HIV infection, although receiving prMOM in the presence of postnatal NVP prophylaxis (Figure 2). These results suggest that NVP prophylaxis may be effective in preventing early transmission of HIV in VLBW infants receiving prMOM; however, as this is the first study reporting on the safety of prMOM in VLBW infants, this observation should be confirmed by larger studies.

## Limitations

Although all infants received prophylaxis, it was carried out inconsistently. Some infants did not receive the first NVP dose immediately after delivery, and in almost half, the weight-based NVP regimen was not adhered to. Notably, only 2/42 infants with suboptimal or no antenatal ART exposure acquired HIV infection in the presence of prMOM. The inconsistencies in the practical implementation of the National PMTCT programme of 2010^19,22^ at clinic and hospital level demonstrate the importance of correct emphasis when training healthcare workers.

Lack of maternal ART may have contributed to HIV transmission in the two HIV-infected infants in this study because neither of the mothers received antenatal or postnatal ART before the infants were diagnosed with HIV infection.

By virtue of the retrospective nature of this study, limitations exist. Reduced infant numbers (*n* = 80) resulting from various exclusions (Figure 1) was the predominant limitation. The largest number of exclusions was for undocumented maternal HIV status (67/690), and no traceable infant HIV-PCR result at ≥ 4 weeks of age (54/219). No long-term follow-up HIV results were available for the 80 included infants, so the overall HIV transmission rate is unknown.

Although the results may be confounded by the 39 deaths prior to 4 weeks of age, the majority of these deaths occurred before 7 days of life (32/39) and are likely to have been immaturity related. However, the possibility of peripartum HIV infection in the late deaths (7/39) cannot be excluded.

Other confounding factors include the possibility of false-negative HIV-PCR tests by 4–6 weeks of age as a result of incomplete viral suppression caused by maternal ART exposure and/or infant prophylaxis.^30^

## Conclusion and recommendations

Viral suppression by antenatal ART followed by infant prophylaxis decreases the risk of MTCT in preterm infants in the presence of rMOM and is likely to protect from life-threatening infection in this group of special – ‘key population’ – patients. Additional personal and public health consequences of breastfeeding such as bonding and long-term successful lactation are of importance to HIV-positive mothers and their children.

## Addendum

The latest National PMTCT programme (2017)^24^ differs from the 2010 programme^19,22^ (Table 2) as follows: Maternal lifelong cART is initiated immediately at HIV diagnosis, irrespective of the CD4 count or HIV staging. Infant PMTCT prophylaxis (drugs and duration) is dependent on maternal factors, with risk classified as low or high. ‘Low-risk’ infants (maternal cART since conception; cART > 4 weeks prior to delivery with a viral load < 1000 copies/mL) receive NVP for 6 weeks, and ‘high-risk’ infants (newly diagnosed maternal HIV; cART < 4 weeks; viral load > 1000 copies/mL) receive dual therapy (NVP plus AZT) for 12 weeks. Breastfeeding is recommended for all HIV-exposed infants (‘low risk’ and ‘high risk’), except for those whose mothers are failing second-line or third-line ART regimens.

## References

[1] Department of Health South Africa. (2015). The 2015 national antenatal sentinel HIV & syphilis survey report [homepage on the Internet]..

[2] Xiao P, Zhou Y, Chen Y, et al. Association between maternal HIV infection and low birth weight and prematurity: A meta-analysis of cohort studies. BMC Pregnancy Childbirth. 2015;15:246 10.1186/s12884-015-0684-z 26450602 PMC4599647

[3] Goga AE, Dinh TH, Jackson DJ, et al. Population-level effectiveness of PMTCT Option A on early mother-to-child (MTCT) transmission of HIV in South Africa: Implications for eliminating MTCT. J Glob Health. 2016;6(2):020405. 10.7189/jogh.06.020405 27698999 PMC5032343

[4] Kourtis AP, Bulterys M Mother-to-child transmission of HIV: Pathogenesis, mechanisms and pathways. Clin Perinatol. 2010;37(4):721–737. 10.1016/j.clp.2010.08.004 21078446

[5] Hoffman RM, Black V, Technau K, et al. Effects of highly active antiretroviral therapy duration and regime on risk for mother-to-child transmission of HIV in Johannesburg, South Africa. J Acquir Immune Defic Syndr. 2010;54(1):35–41. 10.1097/QAI.0b013e3181cf9979 20216425 PMC2880466

[6] Kuhn L, Steketee RW, Weedon J, et al. Distinct risk factors for intrauterine and intrapartum human immunodeficiency virus transmission and consequences for disease progression in infected children. Perinatal AIDS collaborative transmission study. J Infect Dis. 1999;179(1):52–58. 10.1086/314551 9841822

[7] Gupta A, Bhosale R, Kinikar A, et al. Maternal tuberculosis: A risk factor for mother-to-child transmission of human immunodeficiency virus. J Infect Dis. 2011;203(3):358–363. 10.1093/infdis/jiq064 21208928 PMC3071111

[8] Lucas A, Cole TJ Breast milk and neonatal necrotizing enterocolitis. Lancet. 1990;336(8730):1519–1523. 10.1016/0140-6736(90)93304-8 1979363

[9] Schanler RJ Mother’s own milk, donor human milk, and preterm formulas in the feeding of extremely preterm infants. J Pediatr Gastroenterol Nutr. 2007;45(S3):S175–S177. 10.1097/01.mpg.0000302967.83244.36 18185087

[10] Burton R, Giddy J, Stinson K Prevention of mother-to-child transmission in South Africa: An ever-changing landscape. Obstet Med. 2015;8(1):5–12. 10.1177/1753495X15570994 27512452 PMC4934997

[11] Minister of Health v. Treatment Action Campaign (TAC) (2002) 5 SA 721 (CC) [cited 2019 Apr 7]. Available from: https://www.escr-net.org/caselaw/2006/minister-health-v-treatment-action-campaign-tac-2002-5-sa-721-cc.

[12] Kirsten GF, Kirsten CL, Theron A The outcome of very low birth weight infants born to HIV positive women at Tygerberg Hospital. Proceedings of the 26th Conference on Priorities in Perinatal Care. South Africa; 2007 [cited 2019 Apr 7]. Available from: https://www.perinatalpriorities.co.za/proceedings-database/.

[13] Moodley S, Buchmann EJ Outcomes of very low birth weight babies born to HIV positive mothers [unpublished dissertation]. University of the Witwatersrand; 2013 [cited 2019 Apr 7]. Available from: http://wiredspace.wits.ac.za/bitstream/handle/10539/14493/MMED%20Prof%20Buchmann%20Approval%2022102013.pdf;sequence=1.

[14] Delport SD, Dippenaar A HIV and the very low birth weight infant. Presented at the 10th Abbott Round Table discussion. South Africa; 2009 (Unpublished data).

[15] Kirsten GF The impact on the HIV transmission rate of inborn very low birth weight infants born at Tygerberg Hospital of a programme to prevent mother-to-child transmission of HIV which commences at 28 weeks instead of 34 weeks gestation [homepage on the Internet]. Proceedings of the 29th Conference on Priorities in Perinatal Care. South Africa; 2010 [cited 2019 Apr 7]. Available from: https://www.perinatalpriorities.co.za/proceedings-database/.

[16] Tooke L, Horn AR, Harrison MC HIV transmission to extremely low birth weight infants. Pediatr Infect Dis J. 2013;(32)1:36–38. 10.1097/INF.0b013e318270097e 22929171

[17] Levin C, Le Roux D, Harrison M, Tooke L HIV transmission to premature very low birth weight infants. Pediatr Infect Dis J. 2017;36(9):860–862. 10.1097/INF.0000000000001611 28410276

[18] Department of Health South Africa. (2008). Policy and guidelines for the implementation of the PMTCT programme [homepage on the Internet]..

[19] Department of Health South Africa. (2010). Clinical guidelines: PMTCT (Prevention of Mother-to-Child transmission) [homepage on the Internet]..

[20] Jeffery BS, Webber L, Erasmus D Determination of the effectiveness of inactivation of human immunodeficiency virus by Pretoria pasteurisation. J Trop Pediatr. 2001;47(6):345–349. 10.1093/tropej/47.6.345 11827302

[21] Israel-Ballard K, Chantry C, Dewey K, et al. Viral, nutritional, and bacterial safety of flash-heated and Pretoria pasteurised breast milk to prevent mother-to-child transmission of HIV in resource-poor countries. A pilot study. J Acquir Immune Defic Syndr. 2005;40(2):175–181. 10.1097/01.qai.0000178929.15904.95 16186735

[22] Department of Health South Africa. (2010). Guidelines for the management of HIV in children [homepage on the Internet]..

[23] Department of Health South Africa. (2015). National consolidated guidelines for the prevention of mother-to-child transmission of HIV (PMTCT) and the management of HIV in children, adolescents and adults [homepage on the Internet]..

[24] Human immunodeficiency virus infection. In: Standard treatment guidelines and essential medicines list for South Africa. Hospital level paediatrics [homepage on the Internet]. 4th ed. Pretoria, South Africa: The National Department of Health; 2017 [cited 2019 Apr 7], p. 274–275. Available from: http://www.health.gov.za/index.php/standard-treatment-guidelines-and-essential-medicines-list/category/456-hospital-level-paediatrics.

[25] Coutsoudis A, Pillay K, Kuhn L, et al. Method of feeding and transmission of HIV-1 from mothers to children by 15 months of age: Prospective cohort study from Durban, South Africa. AIDS. 2001;15(3):379–387. 10.1097/00002030-200102160-00011 11273218

[26] The Tshwane declaration of support for breastfeeding in South Africa. S Afr J Clin Nutr. 2011;24(4):214.

[27] World Health Organization. Antiretroviral drugs for treating pregnant women and preventing HIV infection in infants: Recommendation for a public health approach [homepage on the Internet]. 2010 [cited 2019 Apr 7]. Available from: https://apps.who.int/iris/bitstream/handle/10665/75236/9789241599818_eng.pdf?sequence=1. 26180894

[28] Department of Health South Africa. (2010). The South African antiretroviral treatment guidelines [homepage on the Internet]..

[29] Kroon M, De Waal R, Horn A, et al. PMTCT guidelines for preterm infants and an interim report of nevirapine trough levels [homepage on the Internet]. Proceedings of the 30th Conference on Priorities in Perinatal Care. South Africa; 2011 [cited 2019 Apr 7]. Available from: https://www.perinatalpriorities.co.za/proceedings-database/.

[30] Sherman GG HIV testing during the neonatal period. S Afr J HIV Med. 2015;16(1), Art. #362:3. 10.4102/sajhivmed.v16i1.362 PMC5843063 29568587

